# Strategies and materials for the prevention and treatment of biofilms

**DOI:** 10.1016/j.mtbio.2023.100827

**Published:** 2023-10-02

**Authors:** Xiaoxia Kang, Xiaoxiao Yang, Yue He, Conglin Guo, Yuechen Li, Haiwei Ji, Yuling Qin, Li Wu

**Affiliations:** School of Public Health, Nantong Key Laboratory of Public Health and Medical Analysis, Nantong University, Nantong, 226019, China

**Keywords:** Anti-biofilms, Strategies, Materials, Prevention biofilms, Treatment biofilms

## Abstract

Biofilms are aggregates of organized microbial growth that function as barriers and create a stable internal environment for cell survival. The bacteria in the biofilms exhibit characteristics that are quite different from the planktonic bacteria, such as strong resistance to antibiotics and other bactericides, getting out of host immunity, and developing in harsh environments, which all contribute to the persistent and intractable treatment. Hence, there is an urgent need to develop novel materials and strategies to combat biofilms. However, most of the reviews on anti-biofilms published in recent years are based on specific fields or materials. Microorganisms are ubiquitous, except in the context of medical and health issues; however, biofilms exert detrimental effects on the advancement and progress of various fields. Therefore, this review aims to provide a comprehensive summary of effective strategies and methodologies applicable across all industries. Firstly, the process of biofilms formation was introduced to enhance our comprehension of the “enemy”. Secondly, strategies to intervene in the important links of biofilms formation were discussed, taking timely action during the early weak stages of the “enemy”. Thirdly, treatment strategies for mature biofilms were summarized to deal with biofilms that break through the defense line. Finally, several substances with antibacterial properties were presented. The review concludes with the standpoint of the author about potential developments of anti-biofilms strategies. This review may help researchers quickly understand the research progress and challenges in the field of anti-biofilms to design more efficient methods and strategies to combat biofilms.

## Graphical abstract

**Figure:**
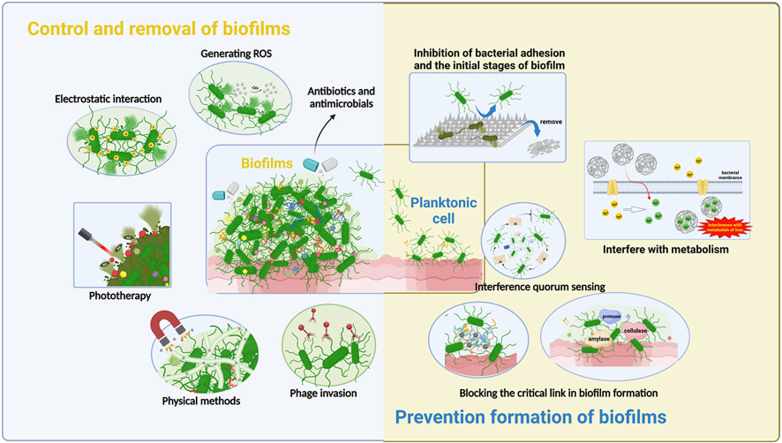

## 1 Introduction

Biofilms are highly organized communities of microorganisms, including bacteria, fungi, and a mixture of strains. They represent a predominant form of microbial life that is ubiquitous in natural ecosystems. The formation of biofilms significantly enhances the survival efficiency of bacteria, rendering them 1000 times more resistant to pressures such as antibiotics, disinfectants, mechanical fluctuations, and physical scavenging compared to their planktonic counterparts [1,2]. For example, the biofilms of *Y. pestis* can enhance their transmission efficiency and withstand fluctuations in the external environment by regulating the feeding behavior of its host flea. This behavior is a consequence of evolutionary selection in response to environmental fluctuations and is referred to as “extended phenotypes” [3]. In addition, bacteria in biofilms can attain a competitive advantage by adopting a slow-growth strategy and occupying a favorable porous microenvironment that promotes the proliferation of diverse bacterial species [4]. Likewise, in iron-deficient environments, *B. subtilis* biofilms deviate from the conventional expansion mechanism by secreting pulcherriminic acid to chelate surrounding Fe^3+^ ions and resist the invasion of alien species [5]. The conclusion can be unequivocally inferred that biofilms significantly enhance the survival ability of planktonic cells during natural selection.

Biofilms have a pervasive impact on all aspects of human life and production. For instance, bacteria can develop highly resistant biofilms within the host organism and medical implant, resulting in chronic and persistent illnesses such as dental caries, sinusitis, osteomyelitis, endocarditis, and even fatality [[6], [7], [8], [9]]. In addition, the formation of biofilms in cooling and drinking water systems presents significant health concerns [10]. The presence of biofilms on metal surfaces can also lead to a reduction in the lifespan of equipment [11]. Finally, as a crucial component of the biosphere, biofilms play an indispensable role in the material cycling [12]. Therefore, uncontrolled biofilms may result in severe ecological consequences. Hence, the persistent contamination and infection resulting from biofilms should not be disregarded in the fields of clinical medicine, food industry, aquaculture, and other industries.

**Fig. 1:**
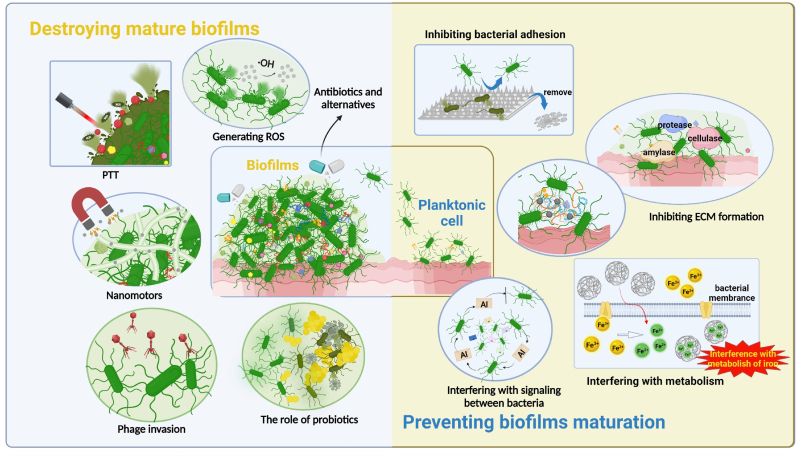
Schematic of anti-biofilms strategies. According to the growth cycle of biofilms, anti-biofilms strategies are divided into two aspects: preventing biofilms maturation and destroying mature biofilms. The prevention of biofilms maturation can be divided into “Inhibiting bacterial adhesion”, “Inhibiting ECM formation”, “Interfering with signaling between bacteria” and “Interfering with metabolism” according to the different stages of biofilms. Strategies to destroy mature biofilms include “Antibiotics and alternatives”, “Generating ROS”, “PTT”, “Nanomotors”, “Phages invasion” and “The role of probiotics”.

The conclusion is that, when confronted with such a formidable adversary, it is imperative to implement impeccable preventive and control measures. This review summarizes the limitations and challenges of current methods for anti-biofilms. The process of biofilms formation is initially introduced to enhance understanding of the “enemy” and develop more targeted strategies for defense and management. Secondly, measures to prevent biofilms formation and intervene in early-stage biofilms are discussed. Thirdly, strategies for controlling and treating mature biofilms are summarized (Fig. 1). The fourth aspect involves the comparison of materials possessing antimicrobial properties to enhance their application in the field of anti-biofilms. Finally, the review concludes by presenting the author's perspective regarding the future direction and expectations of research on anti-biofilms.

The present review provides a comprehensive perspective firmly rooted in the fundamental principles of anti-biofilms, distinguishing itself from other reviews that exclusively concentrate on specific fields or materials. It integrates knowledge from diverse disciplines, including molecular biology, microbiology, materials science, healthcare, and food safety, to analyze strategies for the prevention and control of biofilms. The present review may contribute to a more comprehensive comprehension of strategies targeting anti-biofilms and offer interdisciplinary solutions for addressing biofilms-related issues.

## 2 Formation of biofilms

**Fig. 2:**
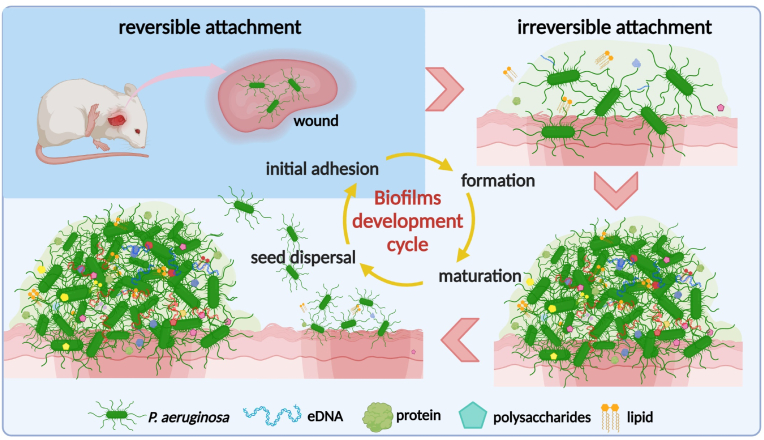
The formation of *P. aeruginosa* biofilms.

A complete biofilms development cycle can be subdivided into 4 stages (Fig. 2). (1) Reversible attachment: planktonic bacteria attached to various environmental surfaces can return to the plankton state. (2) Irreversible attachment: bacteria form a highly organized multicellular community through extracellular polymeric substances (EPS) and extracellular matrix (ECM). By now the bacteria firmly attach to the carrier surface, and it is impossible to return to the single-cell floating state. Then bacteria gather together, grow and reproduce. (3) Maturation: the microbial community continues to grow and merge to form microcolonies with three-dimensional structures which usually look like a mushroom, besides, it can adapt its shape to better suit the surrounding environment. (4) “Seed dispersal” stage: in the late stage, with the increase of the biofilms volume, the bacteria in the inner layer of the biofilms may have growth retardation due to nutrient deficiency and accumulation of toxic metabolic waste. The biofilms undergo a spatial restructuring to enhance the living environment, resulting in the formation of a central cavity within the "mushroom" structure where free seed bacteria are released. Eventually, the top of the biofilms will burst open and disperse free seed bacteria to start new biofilms [13].

In this process, some important substances participate in the formation of biofilms. In the initial stage, bacteria rely on appendages such as flagellum, pili and fimbriae to swim to the surface and settle [14,15]. Then they secrete exopolysaccharides, extracellular DNA (eDNA) and proteins to bind with the surrounding bacteria. In addition, regulatory factors such as c-di-GMP play a crucial role in governing the population dynamics and persistence of biofilms during chronic infections. In a complete biofilms population, they also utilize the quorum sensing (QS) system to sense the surrounding environment to affect the reproduction and decomposition of biofilms [16].

The formation of biofilms facilitates the efficient exchange of genetic material among bacteria and their occupation of distinct ecological niches. Natural biofilms look like an intricate, diverse, and multicultural community akin to our cities [17].

## 3 Preventing biofilms maturation

### 3.1 Inhibiting bacterial adhesion

**Fig. 3:**
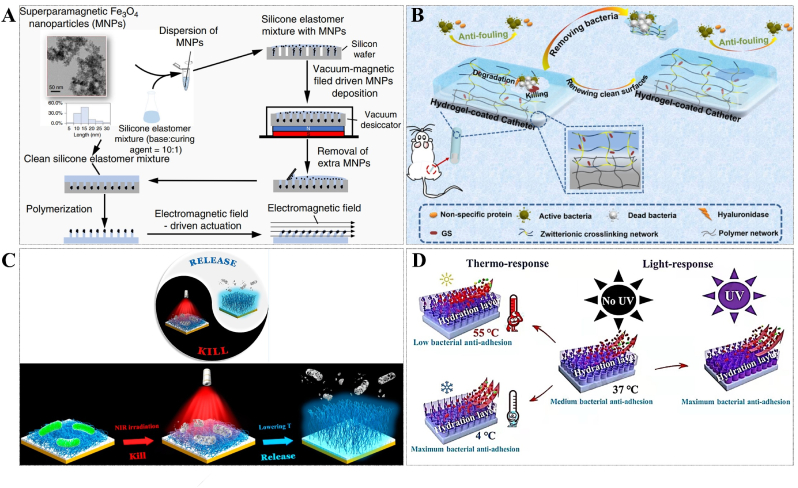
(A) Schematic of the steps for active topography that mimics the ciliary movement of human lung epithelial cells. Reprinted from Ref. [19], Copyright (2020), with permission from Nature Portfolio. (B) Schematic representation of the zwitterionic hydrogel coatings of "Self-Defensive" triggered by acidic biofilms environment. Reprinted from Ref. [21], Copyright (2022), with permission from Amer Chemical Soc. (C) Schematic illustration of thermally triggered antibacterial and antifouling long-lasting coatings. Reprinted from Ref. [22], Copyright (2020), with permission from Amer Chemical Soc. (D) Schematic illustrating the mechanism of the coatings with anti-adhesive effect intelligently controlled by thermo and light. Reprinted from Ref. [23], Copyright (2021), with permission from Elsevier Science Sa.

During the initial stage, bacteria exhibit a limited resistance level owing to the absence of protection provided by developed biofilms. The treatment of biofilms becomes significantly challenging once the bacteria enter the second stage and establish irreversible attachment. Therefore, inhibiting the initial bacterial adhesion is a key way to control the biofilms and avoid a larger crisis. The modification of surface coatings is a widely employed strategy for the prevention of infections associated with implants. Based on the characteristics of antibacterial and antifouling nano-topological structures on cicada and dragonfly wings, Yuan et al. developed a universal method for growing biological nano-daggers on various surfaces, endowing them with high sterilization efficiency [18]. In addition, inspired by the ciliary movement of human lung epithelial cells, which help remove foreign bodies and prevent infection, Gu et al. developed an antifouling surface based on active terrain. Under the action of an electromagnetic field, the micrometer-sized column on the surface of the material could move horizontally in a regular manner and prevent the formation of biofilms of morbific *S. aureus*, *E. coli* and *P. aeruginosa* in the catheter [19] (Fig. 3A). Besides, drawing inspiration from shark skin, Chung et al. designed a Sharklet AF™ surface that interfered with the formation of *S. aureus* biofilms in comparison to the control group [20].

Although antibacterial topological surfaces of antifouling coatings can kill harmful bacteria contact with the surfaces to prevent and reduce bacterial adhesion, their reuse efficiency is often compromised by the accumulation of dead cells and associated debris on the surfaces. Therefore, the presence of coatings that possess both dual antibacterial effectiveness and antifouling activities offers an additional advantage by inhibiting the adherence of deceased cells and debris [24]. Qu et al. developed an efficient smart antibacterial hybrid coating with photothermal bactericidal activity and bacteria release properties. This hybrid coating consists of two functional layers. One is a gold nanoparticle layer (GNPL) composed of gold based nanoparticles (GNPs) with photothermal bactericidal activity and unique micro-nano topography. It offers multiple bacterial contact sites and facilitates localized hyperthermia induced by near-infrared (NIR) radiation. The other layer is the vitamin C-degraded phase transitioned lysozyme film (PTLF) on the surface of GNPL, which acts as a contaminant release layer. The reusability of GNPL-PTLF coatings in multiple kill-release cycles is accompanied by the degradation of the PTLF layer, thereby severely limiting its service life. In addition, the renewal of the coating needs to rely on the role of external vitamin C, which has limitations in many applications [25]. This problem can be solved by using the bacteria's reaction to trigger the renewal of the coating. Zhang and her colleagues constructed a zwitterionic hydrogel coating with [2-(methacryloyloxy)ethyl]dimethyl-(3-sulfopropyl) (SBMA) as the main component and hyaluronic acid (HA) as the crosslinker, as well as the gentamicin sulfate (GS) is grafted to HA by Schiff base bonds. Zwitterionic polymers are electrically neutral and form a barrier against protein adhesion. When a few bacteria escape the coating defenses and attach to the surface, these bacteria will form a weakly acidic microenvironment, resulting in the break of Schiff base bonds and the release of GS rapidly to kill the bacteria. At the same time, the hyaluronidase (HAase) secreted by these bacteria can rapidly degrade the surface and regenerate a new stain-resistant surface [21] (Fig. 3B).

However, the renewal of these two coating materials requires the sacrifice of part of the coating, which affects the long-term performance of the antibacterial coating. Wang and her colleagues developed a smart antibacterial surface with NIR-activated photothermal sterilization and heat-triggered bacterial release properties that can be reused without sacrificing coating materials. The surface deposited tannic acid (TA) and Fe^3+^, and subsequently fixed the poly(N-isopropyl acrylamide) (PNIPAAm) chain, a typical heat-responsive polymer whose solubility phase transition is reversible when the temperature exceeds its lower critical solution temperature (LCST). TA/Fe^3+^ complex is a typical metal ion-phenol network, which shows effective photothermal conversion properties under NIR to kill bacteria. Then the PNIPAAm can easily remove 90% of the attached bacteria from the surface by lowering the temperature [22] (Fig. 3C). However, the formation of such mixed surfaces requires complex equipment and procedures, which limits their practical application. In addition to bactericidal performance and coating renewal ability may be affected by the depth of NIR penetration.

Anti-biofilms are a long-term and unremitting enterprise, requiring antibacterial coatings to not only prevent bacterial attachment and destruction but also actively participate in the prevention of biofilms formation. Zou and his colleagues developed a bifunctional surface composed of an antifouling polymer and a natural anti-biofilms molecule. In this study, quercetin (Qe), a non-toxic flavonoid derived from plants, was employed as a bactericide and demonstrated its efficacy in inhibiting biofilms formation through multiple mechanisms including interference with QS, degradation of the ECM of biofilms, and inhibition of nucleic acid synthesis. In addition, 2-hydroxyethyl methacrylate (HEMA) imparts anti-adhesion properties to the surface and 3-(acrylamido)phenylboronic acid (APBA) provides a binding site for Qe by forming a borate bond. When a few bacteria break through the anti-adhesion layer to proliferate, the acid microenvironment triggers the subsequent release of Qe. Compared to the control group, this dual-functional surface has better anti-biofilms properties, which can guarantee at least 3 d of biofilms prevention without affecting the viability of normal mammalian cells [26].

In addition to considering the antibacterial effect of the coating, it is equally necessary to consider the potential immunological rejection when using it as a medical implant. To prevent overactivation of the immune system, Zwicker analyzed the possible inflammatory effects of macrophages after being exposed to surfaces coated with the antibacterial material poly (hexamethylene) biguanide hydrochloride (PHMB) and dead bacteria. The results showed that the PHMB coating resulted in a high reduction of viable bacteria, and there was no significant difference in cytokine secretion response, indicating good cytocompatibility [27].

Although various surface coatings against bacterial adhesion have been developed, there is still a great demand for antibacterial materials that can adapt to environmental changes such as temperature, pressure, or external wear and tear. Lin et al. integrated the photothermal response strategy with a composite surface, thereby imparting intelligent response capability to the coating for controlling anti-adhesion properties through both thermal and light responses. After 30 times of continuous wear under 27 kPa standard normal stress, the antibacterial rate of the surface against *S. aureus* and *E. coli* only decreased by 1.51%. The statement provides a solid basis for enhancing the durability of antibacterial materials in dynamic environments [23] (Fig. 3D). In addition, the antibacterial coating should react intelligently according to environmental changes and switch functions when necessary. Zhang designed and constructed a pH-responsive titanium-based implant of layer-by-layer surface with dual functions of antibacterial activity and osseointegration promotion. The negatively charged carboxyl group promotes the adhesion, proliferation, and osteogenic differentiation of MC3T3-E1 cells in normal tissues and protects the normal cells from the toxic effects of quaternary ammonium salts (QAS) in the absence of biofilms adhesion on the surface. While the surface is covered by biofilms, anaerobic glycolysis in the anoxic internal environment generates an acidic microenvironment that alters the surface charge at the infected site, thereby triggering further antibacterial action of positively charged QAS. The experimental results of *S. aureus* infected mice confirmed the excellent antibacterial, anti-inflammatory, and bone integration properties of the coating. This adaptive antimicrobial coating strategy offers potential applications in preventing bacterial infections associated with titanium implants [28]. In order to facilitate a more comprehensive comparison of the performance of different coatings, we have compiled their antibacterial mechanisms, primary antibacterial materials, and lifespan in Table 1.

**Table 1:** Comparison of several antibacterial coating materials.

Coating type	Antibacterial mechanism	Major antibacterial material	lifespan^a^	Reutilization	Biocompatibility	Application	Comments	Ref.
Coating with positively-charged ZIF nano-dagger arrays	Electrostatic attraction, physically rupturing cell walls	–	≥2 months	No	–	Surfaces of various materials, such as metal surfaces, poly(methyl methacrylate) (PMMA) plate, polypropylene fiber, silicone, glass, silicone rubber, and filter paper	–	[18]
Magnetically driven active topography	Physical disturbance	Fe_3_O_4_ nanoparticles	≥30 d	Yes	Safety	Medical devices	–	[19]
Sharklet AF™ engineered topography	Engineered surface microtopographies	–	21 d	No	–	Implantable device	–	[20]
Regenerable smart antibacterial surfaces	PTT	GNPL	–	Yes, ≥3 cycles	–	Silicon (Si), PDMS, and SS	The PTLF layer could be degraded by immersion in vitamin C solution to remove the killed bacteria.	[25]
“Self-defensive” antifouling zwitterionic hydrogel coatings	Hydration layer protection and drug sterilization	Zwitterionic poly sulfobetaine methacrylate (PSBMA) and GS	–	Yes	Safety	Medical devices	The coatings of the bacteria-adhering sites would be degraded by HAase secreted by these bacteria and peeled off to remove the bacteria.	[21]
Smart, photothermally activated, antibacterial surfaces	PTT	TA and Fe^3+^ ion	–	Yes, ≥3 cycles	–	Polydimethylsiloxane (PDMS), stainless steel (SS) surface	Releasing dead bacteria by a decrease in temperature.	[22]
Dual-functional anti-biofilms surface	Hydration layer protection, and natural anti-biofilms molecule interferes with biofilms formation	HEMA and Qe	3 d	No	Safety	Implantable device	–	[26]
Smart titanium-containing composite material surface	Hydrated layer protection and electrostatic interaction	QAS and hydrophilic poly(2-hydroxyethyl methacrylate) (PHEMA)	–	Yes	Slight toxicity	Metal materials surfaces	Thermo and light-responsive surfaces can enhance bacterially anti-adhesive property.	[23]
Self-adaptive antibacterial and promoted osseointegration surface	Electrostatic interaction	QAS	–	No	Weak inflammatory response to tissues.	Orthopedic implant	Dual functions of antibacterial and promoted osseointegration.	[28]

a Lifespan refers to the length of time that a surface can effectively anti-bacteria and keep clean before biofilms emerge significantly. The lifespan can vary depending on factors such as the application scenarios, experimental operation, and environmental conditions.

### 3.2 Inhibiting ECM formation

The ECM is a powerful physical and metabolic barrier of biofilms, consisting of polysaccharides, lipids, eDNA, proteins, and so on, which can inactivate antibiotics, enhance the bacterial community, and improve the activity of the drug efflux pump. Dieltjens et al. showed that the inhibition of ECM could increase the susceptibility of bacteria to antibiotics and reduce bacterial adhesion, providing theoretical support for solving the problem of antimicrobial resistance by inhibiting ECM [29].

The eDNA is the predominant compound found in biofilms. Taking *P. aeruginosa* as an example, the formation of "mushroom" biofilms under glucose medium conditions follows a highly organized process. Firstly, the non-motile bacteria constitute the stalk of the mushroom, and subsequently, facilitated by fimbriae, a subset of these bacteria can migrate upwards to form the cap of the "mushroom". The eDNA, bound by fimbriae, can facilitate the aggregation of migrating bacteria along with the high concentration of eDNA outside the stalk. The bacteria subsequently undergo proliferation, leading to the formation of the mushroom cap [13]. Therefore, eDNA plays a crucial role in the formation and structural stability of biofilms. Brown's study found that the exogenous addition of deoxyribonuclease I (DNase I) or restriction enzymes could rapidly degrade eDNA, remove biofilms, and inhibit biofilms reconstitution within 48 h [30]. However, the stability and production cost of this natural enzyme affect its application. Therefore, some researchers have developed more economical and stable artificial simulated nucleases. Chen et al. developed a DNase analog artificial enzyme by adding a variety of cerium (Ce) (IV) complexes on the surface of colloidal magnetic “Fe_3_O_4_/SiO_2_ core/shell”, which exhibit the characteristics of stable, recyclable, and excellent biofilms removal efficiency [31]. Meanwhile, Hu et al. combined artificial enzyme and graphene oxide (GO) with photothermal therapy (PTT) to construct GO-based nitrilotriacetic acid-Ce (IV) composite (GO-NTA-Ce), which effectively eradicated biofilms infections of drug-resistant bacteria [32]. However, its practical application in vivo still needs to be evaluated due to the wide range of non-specific cleavage activity of nucleases.

Genetic and microscopic studies have shown that many different bacteria use proteins as a medium in biofilms formation [[33], [34], [35]]. The amyloid plays a crucial role in the formation of bacterial biofilms, contributing to the initial attachment of bacteria to surfaces, consolidation of bacterial clusters, and maintenance of biofilms structure. Additionally, they may contribute to biofilms resistance against environmental stresses such as antibiotics and immune response [36,37]. Among these, Csg A and Csg B are two critical participants in the synthesis of amyloid curli. Sergei Perov et al. identified structural similarities between fibrillar spine segments derived from Csg A and human pathogenic amyloid. Accordingly, they employed two D-enantiomeric peptides that were initially meant to interfere with Alzheimer's disease to prevent biofilms. It is wondrous that D-enantiomeric peptides prevented Csg A spine fibrillation and reduced biofilms formation of *S*. *typhimurium* [38]. This means that the prevention and control of biofilms can draw insights from the treatment strategies employed in Alzheimer's disease.

Additionally, lectin, a type of carbohydrate-binding protein with multiple glyco-binding sites, plays a pivotal role in the formation of biofilms. This process facilitates bacterial recognition and adhesion while promoting interconnections among bacteria. Among these, lectins Lec B and Lec A are essential compositions of virulence factors and biofilms of *P. aeruginosa*, significantly contributing to its drug resistance [13]. Therefore, the disruption of these proteins' function may potentially hinder the development of drug-resistant bacteria and impede the maturation process of biofilms. Sommer et al. developed a class of Lec B inhibitors with good selectivity and affinity, which effectively blocked the formation of *P. aeruginosa* biofilms in vitro. One of the inhibitors, C-glycoside 7, has an 80–90% inhibition rate on biofilms. Additionally, these inhibitors exhibited good oral bioavailability and excellent drug concentration in the urine and plasma of mice [39]. Besides, Wagner et al. created two diastereoisomeric galactose-derived epoxides to target Cys 62, a key cysteine residue in the carbohydrate-binding domain of Lec A, and produced a fluorescent derivative based on the epoxides for Lec A-specific labeling [40]. This enables the location of bacterial biofilms in infected hosts, making it possible to target pathogens and tissues for treatment. The combination of drugs or antibacterial materials with epoxides offers a promising avenue for advancing biofilms treatment. With the deepening of basic biofilms research, we need to explore more targeted strategies for biofilms treatment to cope with the coming crisis of antibiotic resistance.

### 3.3 Interfering with signaling between bacteria

QS refers to the communication process between cells, which allows bacteria to “communicate” and coordinate their work as a team, ultimately increasing the survival of the entire population in complex environments. In this process, many bacteria can synthesize and release signal molecules called autoinducers (AI). The concentration of AI increases with the accumulating of bacterial density, which can activate the expression of related genes and regulate the biological behavior of bacteria when it reaches certain concentrations [41]. This induction is commonly known as "cell density-dependent regulation of gene expression," as it exclusively occurs once the bacterial population surpasses a specific threshold.

Since animals lack QS systems, blocking QS to prevent the development of biofilms is a safe and viable tactic. To date, many compounds have been discovered to have superior QS system inhibitory action [[42], [43], [44]].

However, the complex and diverse signal regulation mechanisms of QS systems make it extremely difficult to inhibit biofilms with QS inhibitors (QSIs). According to the signal molecules and the sensing mechanism, QS can be divided into three types: oligopeptide QS system of Gram-positive bacteria, Lux I/R QS system of Gram-negative bacteria, and hybrid QS system [13]. For example, the bacterium *P. aeruginosa* possesses three QS systems, namely las, rhl, and pqs [45]. Among them, las and rhl systems belong to the Lux I/R system, which consists of 12-carbon long-chain acyl homoserine lactones (AHL) (N-3-oxododecanoyl-L-homoserine lactone, 3-*O*-C12-HSL) and 4-carbon short-chain AHL (N-butyryl-L-homoserine Lactone, C4-HSL), respectively. The molecules function as signaling molecules, with Las R and Rhl R proteins functioning as their respective receptor proteins. Besides, the pqs system utilizes 2-heptyl-3-hydroxy-quinolone as signaling molecules and Pqs R (Mvf R) as receptor proteins. These diverse and intricate QS systems not only facilitate the growth of bacterial biofilms but also pose significant challenges to biofilm management solely through QSIs. Therefore, multiple strategies often combine with QSI to enhance their effectiveness. The research conducted by Zou resulted in the development of an antibacterial material that utilizes acylase (ACY)'s capability to degrade AHL in Las and Rhl systems through amide bond cleavage, thereby establishing three layers of defense. The outermost layer of HA serves as the primary defense mechanism against initial bacterial adhesion, while the underlying ACY/quaternized chitosan (QCS) layer becomes exposed upon degradation of HA by attached bacteria. The QCS has the ability to eradicate bacteria, while ACY can disrupt the QS system. The material exhibited a remarkable 98% reduction in bacterial adhesion within a span of 4 h, while maintaining a biofilms reduction rate above 90% even after extending the culture time to 28 d [46]. Although this material has a specific and strong effect on bacteria and biofilms of *P. aeruginosa*, the inhibitory effect on Gram-positive bacteria remains to be evaluated.

Moreover, c-di-GMP is a novel second messenger widely distributed among bacteria. It is synthesized by diguanylate cyclase (DGC) enzymes containing the active GGDEF domain and degraded by phosphodiesterase (PDE) containing EAL or HD-GYP active domains [47]. Various metabolic pathways are regulated by controlling the synthesis and decomposition of c-di-GMP. The regulatory principle governing c-di-GMP in most bacteria is that at low concentrations, c-di-GMP enhances bacterial motility and the expression of toxins. While the high concentration of c-di-GMP promotes the expression of adhesion factors and the formation of biofilms. Moreover, the concentration of c-di-GMP within bacteria plays a crucial role in determining whether bacterial cells initiate biofilms formation or remain in planktonic mode as individual cells. Therefore, it represents a promising target for combating bacterial and biofilms infections [48]. Foletti et al. identified the proline-rich peptide Gup-Gup-Nap-Arg from a peptide library, which exhibits selective binding to c-di-GMP and inhibits the growth of *P. aeruginosa* biofilms [49]. However, Gram-positive bacteria exhibit significantly lower levels of GGDEF and EAL domains in comparison to their Gram-negative counterparts, implying that the inhibition of c-di-GMP has a diminished impact on Gram-positive bacteria.

### 3.4 Interfering with metabolism

When transitioning from planktonic cells to biofilms, bacteria may modulate various cellular metabolic pathways to adapt to a non-migratory colony life. These metabolic adaptations play a critical role in biofilms formation. For example, Gelinas' research showed that both *S. aureus* and *E*. *faecalis* require a number of purines to form biofilms, as evidenced by the upregulation of the “de novo purine biosynthesis pathway” during biofilms formation [50]. Additionally, Sinha's study revealed that the biofilms formation of *P. aeruginosa* relies on the ceramidase induced by host lipids to inhibit host wound healing [51]. These altered metabolic pathways could be the targets for the inhibition of biofilms in the future.

**Fig. 4:**
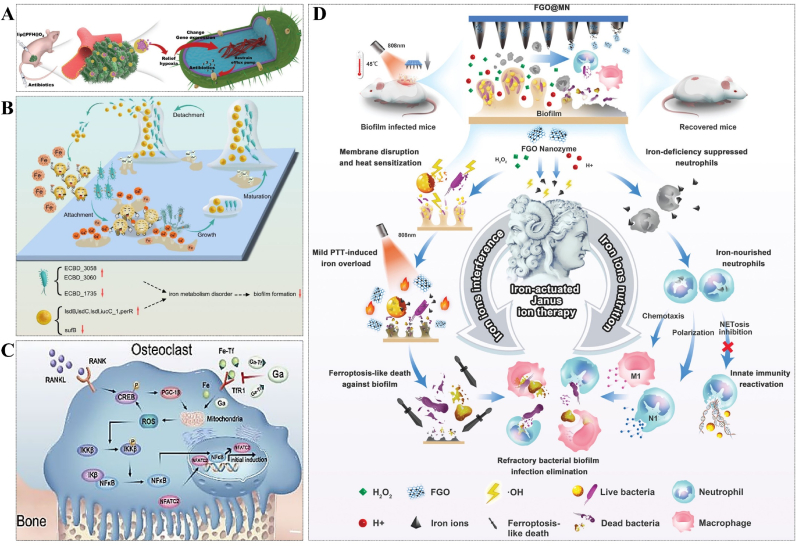
(A) The schematic diagram of treatment of biofilms infection by relieving biofilms hypoxia environment. Reprinted from Ref. [52], Copyright (2020), with permission from Wiley. (B) Ga^3+^ interferes with biofilms formation by disturbing iron ion metabolism. Reprinted from Ref. [53], Copyright (2023), with permission from Springernature. (C) Ga^3+^ inhibits the NF-κB signaling pathway by targeting iron metabolism, then prevents osteoclast differentiation and osteolysis. Reprinted from Ref. [53], Copyright (2023), with permission from Springernature. (D) Schematic illustration of excess iron interfering with biofilms metabolism and activating immune cells. Reprinted from Ref. [54], Copyright (2022), with permission from Wiley-V C H Verlag Gmbh.

Additionally, the formation of biofilms disrupts the delicate balance between internal oxygen consumption and external oxygen supply in bacteria, leading to the creation of an anoxic microenvironment. This is a crucial factor contributing to the resistance exhibited by bacteria coated with biofilms. As a result, relieving hypoxia may be an effective strategy for combating antibiotic resistance in biofilms therapy. Ji's group used perfluorohexane (PFH) as an oxygen carrier in conjunction with liposomes and antibiotics to combat biofilms-related infections. The concentration of antibiotics decreased exponentially with the alleviation of hypoxia, which indicated that modulation of biofilms oxygen metabolism can enhance the bactericidal effect of antibiotics [52] (Fig. 4A).

As one of the essential elements for the survival of most organisms on the Earth, iron ion also plays an important role in the formation of biofilms, which could be evidenced by the fact that siderophores mutant strains of *P. aeruginosa* can only produce flat biofilms rather than mature mushroom biofilms [13,55]. Additionally, to some extent, it has been demonstrated that biofilms can establish an effective defense against the neutrophils and other innate immune cells by competing with them to combine the iron ions. Therefore, it's an effective strategy to realize both biofilms therapy and immune response activation by regulating iron metabolism. Kaneko et al. used gallium (Ga), a non-redox simulant of iron with comparable ionic radius and charge density, to interfere with iron metabolism and biofilms formation, achieving some effect [56]. Similarly, Banin et al. reported that the desferrioxamine-Ga complex could isolate Fe^3+^ and release Ga^3+^ to compete for ferritin binding sites, effectively inhibiting the formation of bacterial biofilms through synergistic effects with gentamicin [57]. However, excess Ga enters the body and may cause damage to the kidneys, leading to kidney failure and even death. This greatly limits the application of Ga in anti-biofilms strategies. The recent evidence suggests that Ga^3+^ can effectively inhibit osteoclast activity [58,59]. After carefully considering the advantages and disadvantages, Ga^3+^ ventured into the realm of anti-biofilms with a fresh identity. The statement suggests that orthopedic patients utilizing biomedical implants will confront two significant therapeutic challenges: aseptic loosening caused by hyperactive osteoclastogenesis and bacterial infection facilitated by the formation of biofilms. Li et al. have successfully developed a biocompatible titanium alloy incorporated with Ga, which demonstrates remarkable capabilities in preventing biofilms formation and shows promising potential in mitigating the risk of aseptic loosening. The statement offers novel perspectives on addressing the clinical challenges associated with implant allocation [53] (Fig. 4B and C). Further investigation by Abouelhassan et al. found that halogenated phenazine antagonized methicillin-resistant *S. aureus* (MRSA) biofilms by influencing iron homeostasis and critical pathways of bacterial surface attachment [60].

In fact, elevated levels of iron ions can also hinder or disrupt the formation of biofilms. Zhu et al. proposed an innovative strategy called "iron-actuated Janus ion therapy (IJIT)" to effectively regulate iron metabolism in both biofilms and immune cells, aiming at combating biofilms formation. The biofilms microenvironmental responsive photothermal microneedle patch (FGO@MN) was developed by growing Fe_3_O_4_ nanoparticles (FNPs) on GO nanosheets and encapsulating them in methacrylated HA needle tips. The combination of GO and FNPs enhances the photothermal properties of the material, while FNPs with peroxidase (PODs) activity generate toxic hydroxyl radicals (·OH) to disrupt the heat shock response (HSR) system in the microenvironment of biofilms. These results allow mild PTT to destroy the biofilms and avoid thermal damage to normal cells. Disruption of the HSR system induces thermal sensitization in biofilms, leading to iron overload in bacterial cells and subsequent iron death. Additionally, this disruption promotes nourishment of surrounding neutrophils to inhibit biofilms. The experiments demonstrated the clearance effect of IJIT on biofilms infection after 15 d [54] (Fig. 4D).

All these phenomena indicate that the bacterial biofilms formation can be inhibited through interfering with bacterial metabolism, thereby presenting a novel approach to combat with biofilms.

## 4 Destroying mature biofilms

### 4.1 Chemical method

#### 4.1.1 Antibiotics and alternatives

Antibiotics with strong and rapid antibacterial effect are widely employed as particular drugs for treating infectious diseases. Different kinds of antibiotics have different therapeutic mechanisms, which can be mainly divided into four categories: (1) interfering with the bacterial cell wall's production, such as penicillins and cephalosporins. (2) affecting the synthesis of bacterial proteins, such as tetracycline, aminoglycoside, macrolides, and chloramphenicol. (3) inhibiting the synthesis of bacterial nucleic acid, such as rifampicin. (4) damaging cell membrane, such as peptides.

**Fig. 5:**
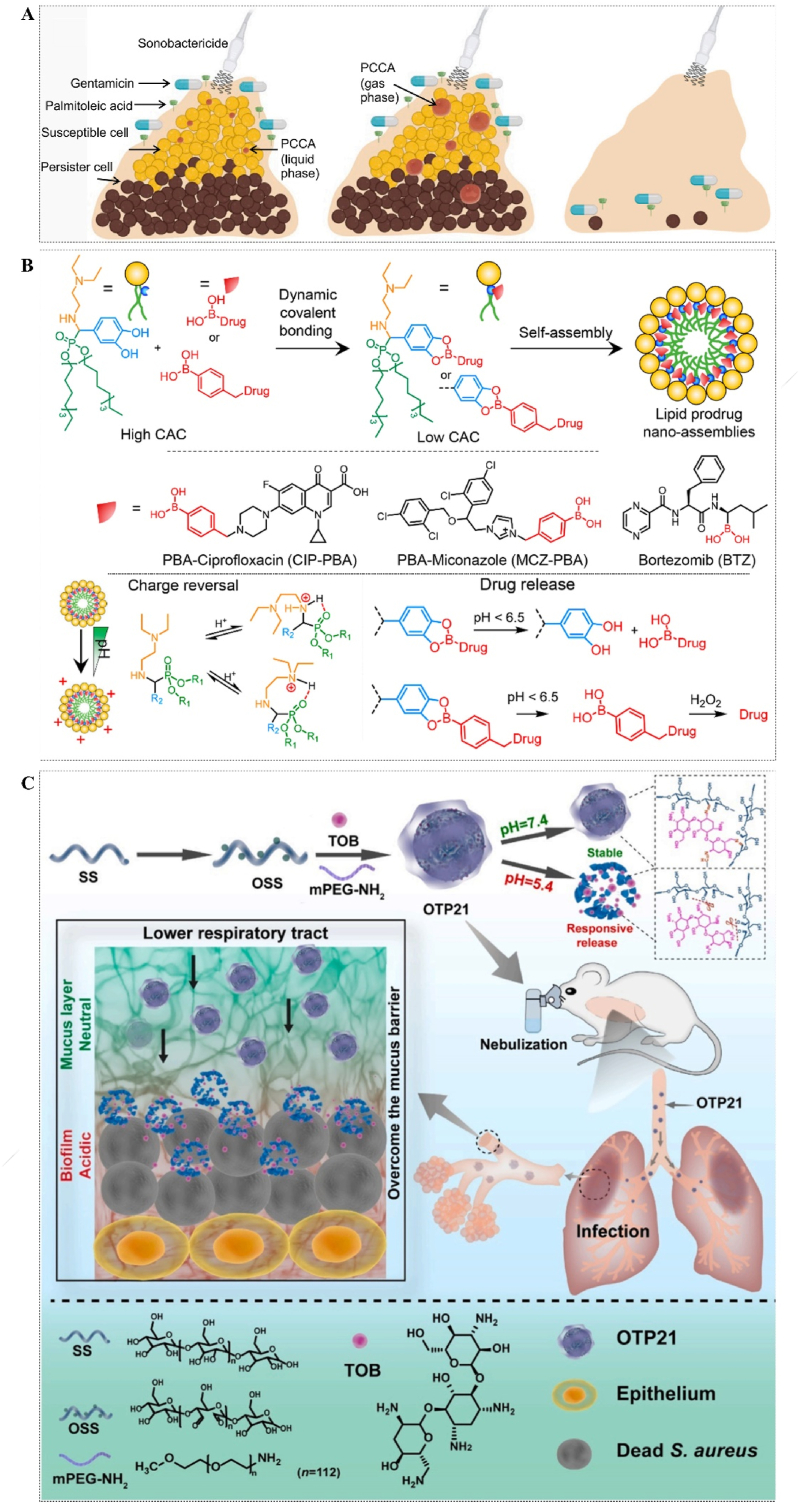
(A) Schematic illustration of PCCA treating biofilms to promote drug penetration. Reprinted from Ref. [61], Copyright (2023), with permission from Cell Press. (B) Schematic diagram of the synthesis of prodrug nanocomponents (LPNAs) by dynamic covalent bonding. Reprinted from Ref. [62], Copyright (2023), with permission from Amer Chemical Soc. (C) Diagram of the synthesis process and mechanism of reactive drug-carrying nanoparticles (OTP). Reprinted from Ref. [64], Copyright (2022), with permission from Elsevier Sci Ltd.

The biofilms that develop at the infection site, however, serve as a robust refuge for bacteria against most antimicrobial agents, thereby necessitating higher drug concentrations and potentially inducing toxic effects on normal cells. In Virginie's study, phase-change contrast agents (PCCA) were introduced to drug-exposed biofilms. The stable liquid PCCA can effectively penetrate the ECM of biofilms and expand into larger gases when exposed to ultrasound. These expanded gases then respond to ultrasonic oscillations, leading to the physical destruction of biofilms and enhanced drug penetration [61] (Fig. 5A). However, this approach may only be effective in addressing superficial biofilms infections of the body, while the treatment of deep tissue biofilms remains a significant challenge. The notion of enhancing drug concentrations as a means to achieve biofilms protection is evidently impractical. The utilization of prodrug nanocomponents holds significant promise in the precise targeting of diseased sites and the on-demand delivery of drugs. Liu et al. reported lipid prodrug nanoassemblies (LPNAs) synthesized by dynamic covalent boric acid between catechol on lipids and boric acid on drug molecules. The LPNAs undergo charge inversion in an acidic microenvironment and selectively release the drug, subsequently eradicating pathogens. Moreover, they exhibit superior efficacy compared to their unencapsulated counterparts in encapsulation and delivery tests involving three model drugs: ciprofloxacin (Cip), bortezomib, and miconazole [62] (Fig. 5B). In order to enhance the drug's efficiency further, they employed the same approach to synthesize antimicrobial hybrid amphiphile (aHA) utilizing DETA NONOate (nitric oxide, NO donor), phenylboronic acid-modified Cip, and 3,4-dihydroxybenaldehyde. The presence of NO can enhance the diffusion of biofilms by modulating the expression of secondary messenger c-di-GMP and bacterial PDE activity, thereby facilitating drug penetration within biofilms. Meanwhile, the aHA can undergo self-assembly into aggregates in aqueous solutions, exhibiting an impressive drug loading capacity of up to 73.8%. This remarkable attribute contributes significantly to its exceptional therapeutic efficacy against peritoneal and subcutaneous infections in mice [63]. Moreover, Ren designed and prepared OTP, a reactive drug-carrying nanoparticle system composed of cross-linked oxidized soluble starch (OSS) and tobramycin (TOB) through Schiff base reaction. Additionally, the super hydrophilic polymer mPEG-NH_2_ was modified to enhance its dispersion and penetration capabilities. The OTP comprises pH-sensitive imine bonds, enabling the release of TOBs upon activation by a slightly acidic biofilms environment, thereby enhancing drug delivery efficiency to the infected area. The hydrophilicity, small volume, and positive surface charge of OTP enhance its penetration efficiency and make it a potential treatment option for lower respiratory tract biofilms infections [64] (Fig. 5C).

Nevertheless, certain bacteria have developed corresponding defense mechanisms to counteract the invasion of antibiotics, including drug permeability disorders, active efflux mechanisms, alterations in drug target sites, and the production of drug-inactivating enzymes. Additionally, the formation of biofilms not only confers protection against drug invasion to bacteria but also facilitates the rapid transmission of resistance genes between bacterial populations. The treatment of biofilms infections with antibiotics poses a significant challenge, necessitating the exploration of alternative approaches.

Instead of antibiotics, antimicrobial peptides (AMPs) are gradually emerging as an alternative antimicrobial drug that does not easily induce drug resistance for the following reasons [65,66]: (1) The bactericidal mechanisms encompass various actions, such as disruption of bacterial cell membranes, modulation of bacterial cell membrane potential, and interference with internal biological processes within bacteria; (2) The broad-spectrum antibacterial activities of AMPs are not reliant on bacteria-specific receptors or enzymes, as they interact with bacterial cell membranes and other targets; (3) The adaptability of AMPs to various environments and targets is facilitated by their intricate structural composition and extensive sequence diversity; (4) The immune system response involves the synergistic interaction of AMPs with other immune cells and molecules to combat bacterial infections.

Engineering peptides based on the sequence of natural peptides have emerged as an excellent choice due to the inaccessibility of natural peptides. Riool et al. synthesized peptides SAAP-276 and SAAP-145 according to the amino acid sequence of human antibacterial peptide LL-37, which demonstrated an excellent inhibitory effect on drug-resistant bacteria and the formation of biofilms without inducing further drug resistance [67]. In addition, Pulido et al. synthesized engineering AMPs based on human RNase Ⅲ, showing great anti-biofilms properties against Gram-negative bacteria [68]. However, AMPs are easily degraded by bacterial and host proteases, so it is crucial to develop a promising drug delivery system to protect its activity. Klodzinska et al. coated the anti-biofilms peptide DJK-5 with a modified HA nanogel. The system showed excellent anti-biofilms properties and excellent biosafety in tests of intravenous and subcutaneous [69].

Essential oils (EOs), delicately crafted by nature, are a profound and treasured gift. They are organic plant extracts made from leaves, petals, roots, seeds, or stems, some of which contain over 300 distinct chemicals, such as alcohols, aldehydes, amides, amines, esters, ethers, heterocycles, ketones, phenols, and terpenes. Therefore, the antibacterial mechanisms of these substances are intricate and not easily susceptible to bacterial resistance. Due to their safety, potent antibacterial properties, and pleasant aroma, they are frequently utilized as natural alternative to chemical preservatives in the food and cosmetics industries. The major components, antibacterial properties, and anti-biofilms mechanisms of several EOs reported in the past two years have been summarized in Table 2 [[70], [71], [72], [73], [74], [75], [76], [77], [78]]. The quality of EOs, however, can be influenced by various environmental factors such as their geographical origin, sunlight exposure, duration of light exposure, and timing of harvest. Additionally, the extraction methods employed also impact the efficacy of these oils [[79], [80], [81]].

**Table 2:** Comparison of antibacterial properties of EO.

EOs	Source	Major Compounds	Pathogen Strains	Dosage/MIC (minimum inhibitory concentration)	Mechanism of anti-bacteria	Effects on biofilms/MBIC (minimal biofilms inhibitory concentration)	Mechanism of anti-biofilms	Safety	Refs.
–	Clinopodium nepeta, Origanum vulgare and Foeniculum vulgare	piperitone oxide, estragole, and p-thymol, respectively	*E. coli* JM109, and its derived antibiotic-resistant cells	0.3–0.966 μL/mL	methylation at both cytosine and adenine residues	–	–	–	[70]
Cedar EO	Cedar wood	e δ-cadinene (36.35%), (Z)-β-farnesene (13.8%), β-himachalene (9.4%),	*M. luteus*	MIC 90: 8.99 μL/mL	–	–	–	–	[71]
Cinnamon EO	–	cinnamaldehyde	*Y. enterocolitica*	0.625 mg/mL	altering bacteria cell membrane structure, stability, and osmotic function	0.078 mg/mL	interfering with the QS system by inhibiting AHL production	–	[72]
Clove essential oil (CEO) and Oregano essential oil (OEO)	–	eugenol and carvacrol, respectively	*S. Derby*	0.8 mg/mL and 0.2 mg/mL, respectively	–	At 1/8 MIC, the inhibition rate of CEO and OEO on the biofilms formation was 90.29% and 48.79%.	suppressing the metabolic activity and the production of the extracellular polysaccharide	–	[73]
Composite EO	–	cinnamaldehyde, carvacrol, and eugenol	*E. coli* and *S. aureus*	7.94 μL/mL	destroying bacteria cell wall and cell membrane integrity, and retarding the respiratory chain	The biofilms of *S. aureus* decreased by 11.19%, 15.32%, and 18.09%, and that of *E. coli* decreased by 14.25%, 21.72%, and 22.37% after exposure (0 h) to EOs at 0.5 MIC, 1 MIC, and 2 MIC, respectively.	–	–	[74]
J. intigrimma, J. roseae and J. gossypifolia EO	leaves of Jatropha	diterpenes	*E. coli*	5 mg/mL, 5 mg/mL, and 2.5 mg/mL, respectively	–	31.25 μg/mL, 250 and above 1000 μg/mL, respectively	disturbing adhesion proteins FimH	–	[75]
Propolis essential oil (PEO)	complex material that honey bees collect from resinous and balsamic material	β-himachalene (13.94%), α-curcumene (11.28%), α-bergamotene (4.5%)	*S. mutans*	0.625 μL/mL	–	1/4 MIC or above can significantly reduce the biofilms biomasses compared to the control group (p < 0.05).	membrane permeability, releasing lactic dehydrogenase (LDH) and calcium ions; inhibiting bacterial proliferation; suppressing the activity of glucosyltransferases (GTFs) to reduce the production of extracellular polysaccharides and alleviate bacterial adherence	IC10 value of PEO on HOECs was 1.299 μL/mL, which was significantly higher than double the MIC.	[76]
Thymus x citriodorus EO	Thyme species	monoterpenes (72.6%), being geraniol (27.5%), 1,8-cineole (16.3%), and thymol (9.2%);	*C. acnes* and *S. epidermidis*	0.06%	–	0.12%–0.24%	–	affecting cellular viability in a dose-dependent manner.	[77]
Thymus zygis subsp. gracilis EO	Lamiaceae	*p*-cymene (25.98 ± 0.07%), thymol (22.64 ± 0.06%), carvacrol (21.28 ± 0.06%), and γ-terpinene (11.01 ± 0.03%)	*L. monocytogenes*	0.02% (v/v)	–	0.02% (v/v)	–	–	[78]

#### 4.1.2 Generating ROS

Reactive oxygen species (ROS) are chemically active atoms or groups containing oxygen, such as singlet oxygen (^1^O_2_), hydrogen peroxide (H_2_O_2_), ·OH, ozone (O_3_), NO, and so on. ROS can destroy the structure of the cell membrane by inducing unsaturated fatty acids in lipids to undergo peroxidation reactions, and further damage nucleic acids, proteins, as well as other biological macromolecules.

**Fig. 6:**
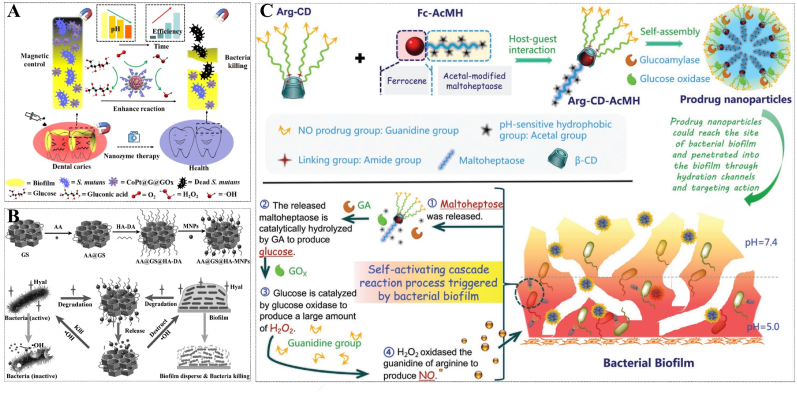
(A) Anti-biofilms mechanism of cascade reaction hybrid PODs nano-enzyme (CoPt@G@GOx) for oral biofilms therapy. Reprinted from Ref. [83], Copyright (2022), with permission from Tsinghua Univ Press. (B) Schematic representation of synthesis process and mechanism that biofilms-induced on-demand ROS release delivery system "AA@GS@HA-MNPs". Reprinted from Ref. [84], Copyright (2016), with permission from Wiley-V C H Verlag Gmbh. (C) Schematic illustration for anti-biofilms therapy with Arg-CD-AcMH that cascade catalytic NO release. Reprinted from Ref. [85], Copyright (2022), with permission from Wiley-V C H Verlag Gmbh.

ROS can be produced in several methods to induce bactericidal and anti-biofilms effects. The process of photodynamic therapy (PDT) involves the excitation of a photosensitizer by laser irradiation at a specific wavelength, which subsequently transfers energy to surrounding oxygen molecules, leading to the generation of ROS. Chen et al. developed bimetallic metal-organic frameworks (MOFs) PCN-224 (Zr/Ti) that significantly stimulated the generation of ROS under visible light and exhibited effective antibacterial activity [82]. The anoxic microenvironment of the biofilms, however, can significantly diminish the efficacy of PDT. PODs-active nano-enzymes that catalyze the conversion of zymolyte into ROS in an oxygen-independent manner-are commonly utilized in anti-biofilms techniques. Dong's group designed a cascade reaction PODs nano-enzyme “CoPt@graphene (G) @glucose oxidase (GOx) (CoPt@G@GOx)”. The GOx enzyme oxidizes glucose to generate H_2_O_2_, which acts as a substrate for the POD mimicking "CoPt@G" catalyst, resulting in the production of highly reactive ·OH species. Additionally, the magnetic properties of "CoPt@G@Gox" facilitate the enrichment of nanoparticles (NPs), leading to approximately 6 × 10^4^ times enhanced antibacterial activity in the presence of a magnetic field [83] (Fig. 6A). This strategy demonstrates a favorable efficacy in the management of oral diseases caused by biofilms. However, the presence of glucose as a vital energy source poses a new challenge in preventing the premature release of ROS during infection treatment. Here, Ji et al. constructed a targeted on-demand drug delivery system “AA@GS@HA-MNPs” by encapsulating ascorbic acid (AA) in graphene-mesoporous silica nanosheet (GS) and then modifying magnetic NPs (MNPs) and HA onto the surfaces of hybrids. Meanwhile, the MNPs were precoated with vancomycin. Upon arrival at the infected site, the HA coating of "AA@GS@HA-MNPs" is degraded by bacterial-secreted HAase, facilitating subsequent release of AA that can be catalyzed by MNPs to generate ·OH in situ. The combination of G's exceptional PTT properties with this system demonstrates a synergistic antibacterial and anti-biofilms activity against both Gram-positive and Gram-negative bacteria [84] (Fig. 6B).

NO is an attractive antimicrobial agent that can both disperses bacterial biofilms and prevents them from developing drug resistance. Additionally, NO can accelerate wound healing by boosting the creation of collagen and the production of myofibroblast during skin reconstruction [[86], [87], [88]]. Therefore, it gradually occupies an important position in the infection treatment of biofilms. Taking the limited diffusion range and rapid degradation of NO into account, it is also imperative to devise diverse strategies for continuous NO delivery to the ECM. Jin-Wook's team has developed a “polyethyleneimine/diazoate-doped PLGA” that can bind to biofilms substrates through electrostatic interaction and promote NO delivery to wounds infected by MRSA biofilms. The release time of NO in the simulated wound fluid was prolonged by over 4 d, while the biofilms were dispersed in diabetic mice, resulting in accelerated wound healing compared to the control group on the 8th day post-injury [89]. To achieve a more precise release of NO at the site of wound infection, Shi and her colleagues opted for β-cyclodextrin (β-CD) modified by NO donor L-Arginine (L-Arg) and maltoheptaose (MH) modified by linear ferrocene terminated acetal as the framework molecules to synthesize amphipathic supramolecular Arg-CD-AcMH. Subsequently, glucoamylase (GA) and GOx were incorporated through electrostatic interaction. The NPs, upon reaching the surface of bacterial biofilms through hydration channels, encounter a weak acid environment (pH 5–6) that triggers the cleavage of the acetal bond and subsequent release of MH. Subsequently, MH undergoes hydrolysis by GA to generate glucose, which is then catalyzed by GOx to produce a significant amount of H_2_O_2_. This abundance of H_2_O_2_ activates L-Arg and leads to a substantial release of NO [85] (Fig. 6C). The sequential activation of ROS cascades enables precise localization of ROS release at the infection site, thereby minimizing damage to normal cells. Additionally, the utilization of NO disrupts biofilms formation and facilitates wound healing.

### 4.2 Physical method

#### 4.2.1 PTT

The PTT technique induces local hyperthermia by employing non-invasive light irradiation and utilizing photothermal agents with high conversion efficiency. The bioactive substances, such as nucleic acids or proteins, are rendered inactive, compromising the integrity of bacteria and the structure of biofilms, thus augmenting the sterilization efficacy. It has benefits including tissue penetration, spatiotemporal controllability, and minimal invasiveness. Various materials such as Graphene, carbon quantum dots, metals, polydopamine (PDA), organic π-conjugated molecules and so on all exhibit certain PTT properties under NIR irradiation [[90], [91], [92], [93]]. The Food and Drug Administration (FDA) has granted clinical approval for the use of commonly employed molecules, such as indocyanine green (ICG) and methylene blue (MB).

The precise delivery of these PTT materials to the infected site poses a challenge. The PDA-ICG-NPs were prepared by Gao et al. using polyethylene glycol (PEG) and poly(beta-amino ester) (PAE), a pH-responsive mixed shell polymer, to encapsulate ICG-coated PDA. The presence of electrostatic interaction enables the transportation of PDA-ICG-NPs in the bloodstream, facilitating their targeted delivery, penetration, and accumulation on biofilms [94].

**Fig. 7:**
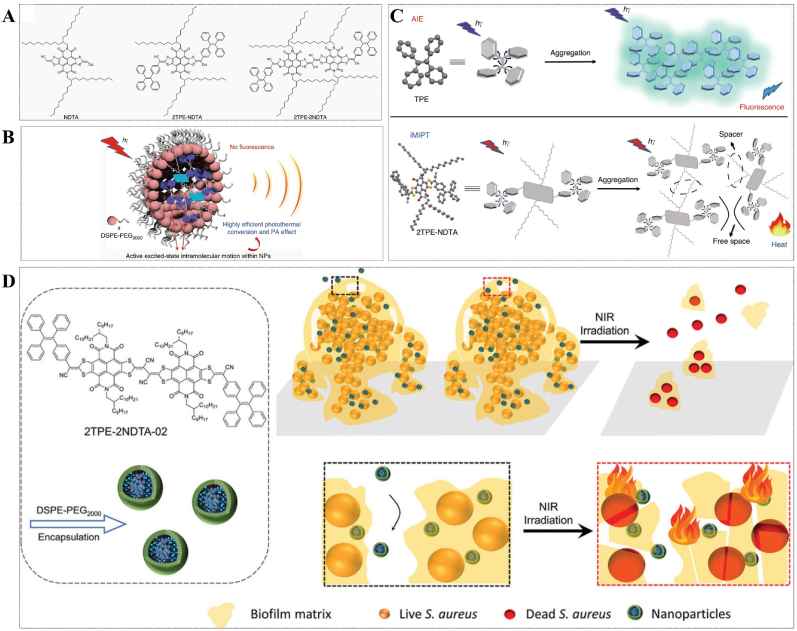
(A) Molecular structures of iMIPT nanoparticle precursor and two kinds of iMIPT nanoparticle. Reprinted from Ref. [95], Copyright (2019), with permission from Nature Portfolio. (B) 2TPE-NDTA-doped NP exhibits an outstanding iMIPT peculiarity. Reprinted from Ref. [95], Copyright (2019), with permission from Nature Portfolio. (C) Comparing the mechanisms of AIE and iMIPT. Reprinted from Ref. [95], Copyright (2019), with permission from Nature Portfolio. (D) An efficient anti-biofilms strategy for PTT based on the iMIPT mechanism. Reprinted from Ref. [96], Copyright (2021), with permission from Royal Soc Chemistry.

However, planar molecules like ICG exhibit strong face-to-face π-π stacking interactions in the aggregation state, leading to non-radiation decay and insufficient radiation decay. In order to effectively control molecular mobility in the aggregate/solution state and enhance the molar absorption rate, Tang's research team proposed an innovative concept for molecular design. The intramolecular excited state motion facilitated the successful synthesis of nanoparticles with excellent PTT characteristics induced by intramolecular motion (iMIPT) [95] (Fig. 7A–C). In subsequent studies, they prepared TN NPs with higher photothermal conversion properties based on iMIPT NPs, which successfully destroy mature *S. aureus* biofilms under NIR irradiation (808 nm, 1 W cm^−1^) without hazards in the dark [96] (Fig. 7D).

**Fig. 8:**
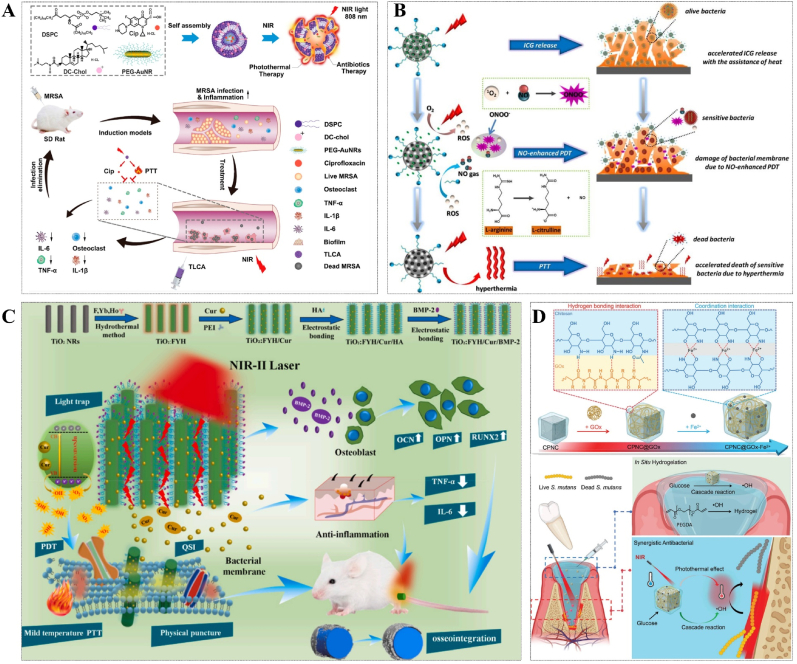
(A) Composition, structure, and mechanism of antibiotics and PTT combined antibacterial platform triggered by NIR. Reprinted from Ref. [97], Copyright (2023), with permission from Amer Chemical Soc. (B) AI-MPDA nanoparticles' biofilms removal mechanism is based on low-temperature PTT and NO-enhanced PDT. Reprinted from Ref. [98], Copyright (2020), with permission from Amer Chemical Soc. (C) Schematic diagram of the process for rapid biofilms elimination of Ti implants at mild temperatures assisted by NIR Ⅱ. Reprinted from Ref. [99], Copyright (2020), with permission from Elsevier Sci Ltd. (D) Schematic diagram of the synthesis process and mechanism of a cascade nanoreactor CPNC@GOx-Fe^2+^ with photothermal and wound protection properties. Reprinted from Ref. [100], Copyright (2023), with permission from Wiley-V C H Verlag Gmbh.

The use of PTT alone, however, requires temperatures above 70 °C, while excessively high temperatures can have detrimental effects on healthy tissue. In this case, Zhao et al. developed a cationic heat-sensitive liposome by coupling antibiotics with gold (Au) nanorods for the treatment of MRSA-induced osteomyelitis. The NPs with a positive charge can effectively penetrate the biofilms, enhancing their permeability and facilitating increased antibiotic access to bacteria. More than 80% of the antibiotics were rapidly released at a local temperature of 50 °C under 808 nm laser irradiation, resulting in the destruction of most of the biofilms [97] (Fig. 8A). Additionally, the combined application of PDT and PTT is frequently employed in numerous research studies for biofilms treatment. For instance, Yuan et al. proposed AI-MPDA, an integrated nanoplatform for light therapy comprising mesoporous polydopamine (MPDA), ICG, and L-Arg. The NO released by L-Arg combined with low-temperature PTT (≤45 °C) effectively eradicates bacteria and disrupts biological membrane structures within a few minutes of NIR irradiation [98] (Fig. 8B).

The light undergoes gradual attenuation as it propagates through human tissues due to internal dispersion, reflection, and absorption. Therefore, the applicability of NIR-Ⅰ (808 nm) is limited in comparison to NIR-Ⅱ (1000–1700 nm). Chu's team has developed a NIR-II PTT anti-biofilms system consisting of curcumin (Cur) and co-doped rare earth ions Ho, Yb, and F titanium dioxide (TiO_2_) NRs. This system overcomes the limitation that TiO_2_ cannot be activated by NIR-II light and enhances the photocatalytic activity of TiO_2_. Cur endows the material with QS inhibition and anti-inflammatory properties. The system effectively prevents mild implant infection in the absence of light irradiation and efficiently eradicates biofilms at 45 °C under NIR-II irradiation. After penetrating 1.2 cm of pork, the power output of the 808 nm laser decreases to less than 0.03 W, while the power output of the 1060 nm laser remains approximately at 0.05 W even after penetrating through a thickness of 2.2 cm [99] (Fig. 8C).

The utilization of appropriate dressings is essential in addition to PTT and other treatment methods for effective eradication of wound biofilms, ensuring protection against secondary injury caused by residual bacteria. For this purpose, Chen et al. developed a chitosan-modified palladium nano-cube (CPNC) based photothermal cascade nanoreactor (CPNC@GOx-Fe^2+^) incorporating GOx and Fe^2+^, which effectively catalyzes the oxidation of glucose to generate highly reactive ·OH. Besides, the photothermal characteristic of CPNC had a synergistic bacteriostatic effect on *S. mutans*. At the same time, the continuous generation of ·OH initiates the radical polymerization of poly(ethylene glycol) diacrylate (PEGDA), leading to the in situ formation of hydrogels, which create a moist and sterile environment for wound healing. The proposed approach presents a promising solution for the treatment of open wound infections. However, its application is restricted to superficial bacterial biofilms infections due to the limited tissue penetration ability of NIR [100] (Fig. 8D).

#### 4.2.2 Nanomotors

In the past few years, nanomotors have been used to destroy bacterial biofilms due to their active motion and high permeability. Among them, magnetic nanomotors based on Fe_3_O_4_ have been widely used to artificially destroy the membrane, which significantly improves the penetration ability and bactericidal effect of drugs on biofilms. However, the development of MNPs with targeted mobility is challenging. Stanton et al. utilized the non-pathogenic magnetotactic bacteria (MSR-1) and drug-loaded mesoporous silica microtubules to construct biological hybrids that could be directed into mature *E. coli* biofilms. Then the acidic microenvironment of the biofilms could trigger the release of the antibiotic Cip, ensuring efficient drug delivery [101].

**Fig. 9:**
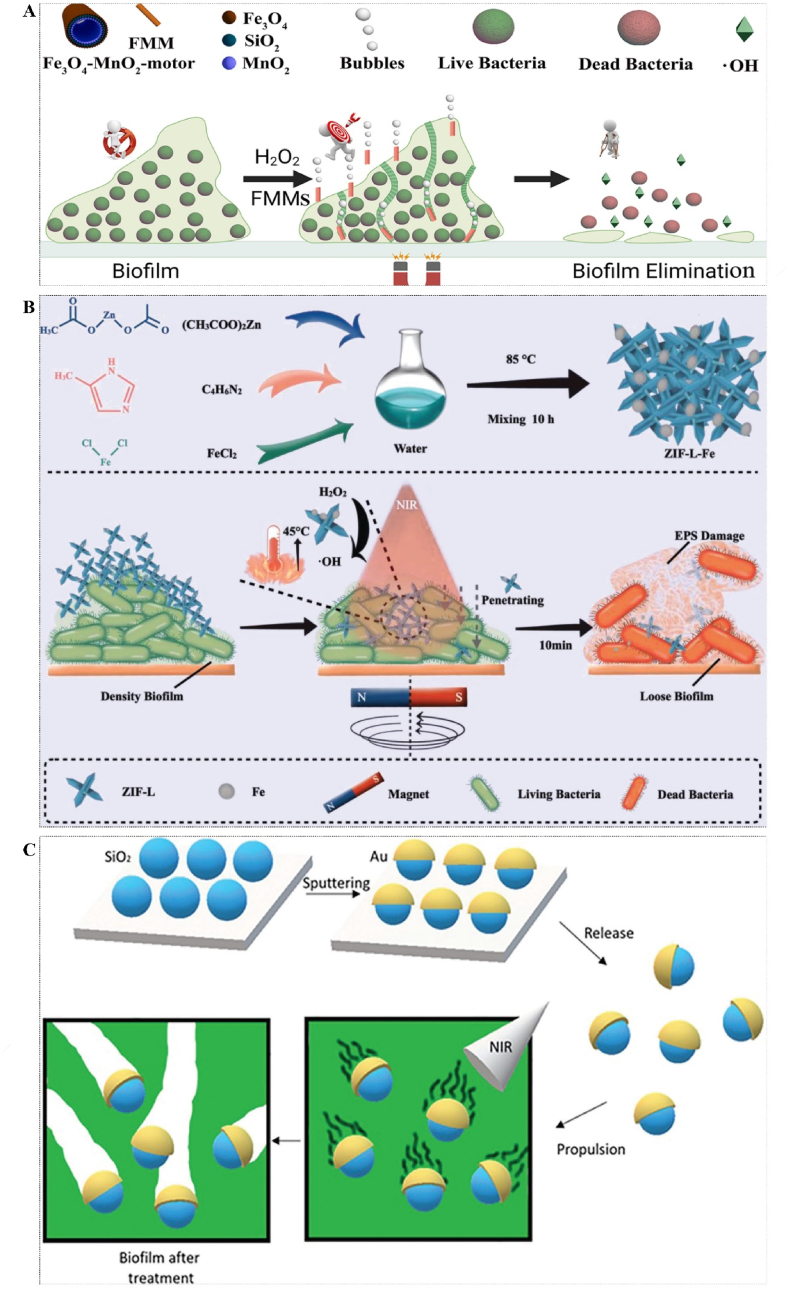
(A) Schematic of a multifunctional nanomotor driven by magnetic and dye. Reprinted from Ref. [102], Copyright (2022), with permission from Elsevier. (B) Synthesis process and mechanism of magnetic cloud bomb. Reprinted from Ref. [103], Copyright (2023), with permission from Wiley-V C H Verlag Gmbh. (C) Schematic diagram of the mechanism of a self-propelled mesoporous SiO_2_/Au nanomotor driven by NIR light. Reprinted from Ref. [104], Copyright (2023), with permission from Wiley.

In addition, the magnetically driving NPs to drill biofilms still have many defects, such as slow motion and uncontrolled direction. Therefore, Ji et al. developed a magnetically controlled multifunctional micromotor system by using MNPs and mesoporous silica with MnO_2_ as catalysts and H_2_O_2_ as fuels. When MnO_2_ contact with H_2_O_2_ in the mesopore, a huge number of oxygen microbubbles could be immediately released, acting as a powerful propellant to propel the NPs move through the biofilms. Meanwhile, H_2_O_2_ was transformed into a highly toxic ·OH to kill bacteria due to the presence of magnetic Fe_3_O_4_. This strategy combines powerful mechanical damage, ROS, and precise magnetic guidance to achieve a short time of sterilization and anti-biofilms effect [102] (Fig. 9A).

Still, the destruction effect of the biofilms is also influenced by the nanomotors' structure and dispersion. Sharp-shaped NPs are more likely to breach bacterial membranes and cause cytoplasmic leakage compared to blunt particles. Inspired by traditional cloud bombs, Qian et al. have modified the structure of zeolitic imidazolate framework (ZIF) from a sheet-like form to a flower-like configuration with sharp edges, known as ZIF-L-Fe. This modification facilitates enhanced penetration of biofilms through the application of magnetic force and promotes improved dispersion of Fe NPs. Additionally, it possesses PTT and PDT properties, enabling the eradication of MRSA at low temperatures (42–45 °C) while effectively catalyzing H_2_O_2_. In vitro or in vivo experiments conducted on mice skin wounds demonstrated that the synthesized MNPs exhibited potent antimicrobial effects against MRSA and biofilms [103] (Fig. 9B).

Although magnetically driven nanoparticles offer an ideal means of physically destroying biofilms, the loading and regulation of the magnetic field pose significant challenges that are constrained by various factors, including material limitations and biofilms location. Maric initially proposed a self-propelled nanomotor composed of mesoporous SiO_2_/Au, which is driven by NIR light. In this investigation, mesoporous silica nanoparticles were coated with a thin layer of Au to create asymmetric nanomotors. The mesoporous SiO_2_/Au nanomotors exhibited remarkable self-propulsion performance, ranging from a few μm/s to approximately 100 μm/s, which could be finely tuned by adjusting the laser power. Additionally, in conjunction with the PTT effect of Au, the SiO_2_/Au nanomotors exhibit enhanced penetration through the ECM and dispersion of biofilms. Following laser irradiation for 30 s and 3 min, there was a respective increase in the eradication rate of biofilms by 20% and 71%, respectively [104] (Fig. 9C). The light-driven nanomotor exhibits superior remote controllability compared to magnetically driven nanomotors. However, it is imperative to consider the targeting of biofilms and potential damage caused by light and heat to normal tissues.

### 4.3 Biological method

#### 4.3.1 Phages invasion

Phages are frequently recommended as an alternative to antibiotics for treating biofilms since they are affordable, targeted, and do not disrupt the normal microbiota [105]. Some phages also possess the ability to degrade polysaccharides and rapidly destroy the integrity of the membrane [106,107]. Significantly, Cano et al. demonstrated the effectiveness of phages in the clinical treatment of recurrent *Klebsiella pneumoniae* infection on the false knee by applying the intravenous phage therapy. After treatment, the local infection symptoms were dramatically reduced, and the false knee function gradually improved without related adverse events [108]. This is a significant attempt of phages to move from the experimental to the clinical stage. Engineering bacteriophages may be promising candidates for the inhibition of pathogenic bacteria and biofilms in the future [109,110].

The early stage of phage therapy, however, involves extensive preparatory work that necessitates time and precise laboratory technical support, thereby augmenting the intricacy of treatment. In addition, the human immune system may elicit an immunological response to the phages, thereby diminishing their therapeutic efficacy and potentially inducing allergic reactions. Further investigations are warranted to substantiate the long-term effectiveness and safety of phage therapy.

#### 4.3.2 The role of probiotics

Interestingly, not all biofilms exert a negative impact on human production and life. For instance, some biofilms play an essential role in delaying food spoilage, inhibiting toxic microorganisms, optimizing fermentation, and improving the yield and quality of food fermentation. Moreover, *E. faecium* and *P. pentosaceus* isolated from fermented fish and chickens have potential antibacterial and anti-biofilms effects on foodborne pathogenic bacteria, including *B. cereus*, *S. enterica* and *E. coli* [111]. Besides, *T. halophilus*, which is used to season salty fermented dishes, also possesses anti-biofilms activity against *S. typhimurium* and *S. aureus* [112]. These aforementioned methods employ beneficial bacteria to fight harmful bacteria, exhibiting excellent potential in the food industry. More importantly, this method has little environmental pollution, lasting effects, low cost, safety for people and livestock, and is not easy to produce resistance.

## 5 Raw materials with bactericidal and anti-biofilms potential

In addition to antibiotics, certain materials are extensively utilized in the field of antibacterial and anti-biofilms applications owing to their inherent properties. Here, we provide a comprehensive overview of the antimicrobial mechanisms and diverse applications associated with these commonly employed materials (Table 3).

**Table 3:** Antimicrobial mechanism and application of common materials.

Material	Antibacterial mechanism	Application	Bacterial type	Antibacterial adjuvant and antibacterial mechanism	Biocompatibility	Ref.
Au	Physical destruction of the biofilms by light-driven motion	–	*P. aeruginosa*	–	–	[104]
Au nanorods	PTT	Ablation infected osteomyelitis	MRSA	Cip (antibiotic)	Cell viability was >80%.	[97]
Au NPs		Treatment of subcutaneous abscess	*S. aureus*	PHMB (electrostatic interaction)	Cell viability was >90%.	[113]
Fe^2+^	·OH is produced through the Fenton or Fenton-like reaction	Protecting tooth extraction wound	*S. mutans*	CPNC (PTT)	No significant cytotoxicity, and hemolysis rates <5%.	[100]
Fe_3_O_4_	Magnetically driven physical destruction	Treatment of skin and soft tissue infection	MRSA	ZIF (physical destruction of the biofilms by a flower-like structure with sharp edges)	Cell viability was >90%.	[103]
		Elimination of microchannels biofilms	*S. aureus*, MRSA and *P. aeruginosa*	H_2_O_2_ (dye drives physical destruction of the biofilms)	No significant cytotoxicity.	[102]
	POD-like activity catalyze the generation of·OH	Wounds and subcutaneous implant-associated biofilms infection treatment	*S. aureus* and *E. coli*	GO (PTT)	No significant cytotoxicity.	[54]
Ga^3+^	Interfering with Fe^3+^ metabolism of bacteria or biofilms	Orthopedic implants Ti–Ga alloys	*S. aureus* and *E. coli*	–	No significant cytotoxicity.	[53]
Pt	POD-like activity catalyze the generation of ·OH	Therapy of biofilms-induced periodontitis	*S. aureus*	–	Cell viability was >90%, and hemolysis rates were <5%.	[114]
Ce (IV)	DNase activity, degrades eDNA	Treatment of subcutaneous abscess	*S. aureus*	GO (PTT)	Cell viability was >80%.	[32]
Snitrosoglutathione (GSNO)	Release NO due to GSNO could be disrupted by heat, light, and some metal ions	Therapy of infected wounds	*E. coli* and *S. aureus*	–	Cell viability was >90%.	[88]
L-Arg	Release NO due to oxidization of H_2_O_2_	Treatment of infected wounds.	*E. coli* and *S. aureus*	–	–	[85]
NONOate	Release NO in an acidic microenvironment	Alleviate inflammation in a subcutaneous infection	*S. aureus*	Cip (antibiotic)	No significant cytotoxicity.	[63]
QSC	Electrostatic interaction	Coating	*P. aeruginosa*	ACY (QSI)	Cell viability was >95%.	[46]
QAS	Electrostatic interaction	Coating applied on the surface of orthopedic implant materials	*E. coli* and *S. aureus*	–	Hemolysis rates <0.1%.	[28]

## 6 Conclusions and future perspectives

Biofilms are bacterial populations surrounded by ECM that operate as a barrier against external antimicrobial agents. Developing new antibacterial drugs and strategies has become an urgent demand. We summarize the limitations and development prospects of the above strategies (Table 4).

**Table 4:** Limitations and prospects of anti-biofilms strategies.

Strategies			Advantages	Disadvantages	Application						
					Superficial wound	Deep tissue	Medical implant	Food packaging	Food preservation	Instrument and equipment	Environmental governance
Preventing biofilms maturation	Inhibiting bacterial adhesion		Tackling the root of problems	Limited permanence; immunological rejection; uncontrollable and non-selective			✓	✓		✓	
	Inhibiting ECM formation		Cutting the key link	In addition to eDNA, other EPS lack sufficient research; safety and efficacy need to be evaluated; highly specific and may not be effective for other biofilms	✓	✓	✓				
	Interfering with signaling between bacteria		Cutting the key link; less toxic side effects	Effective for specific biofilms; complex signal transduction mechanism; dose assessment is difficult	✓	✓	✓				✓
	Interfering with metabolism		Cutting the key link	Affect normal metabolism in the body; toxic and side effects need to be evaluated	✓	✓	✓				✓
Destroying mature biofilms	Chemical methods	Antibiotics and alternatives	Usual strategy; clear mechanism	Antimicrobial resistance; drugs are difficult to penetrate biofilms	✓	✓	✓				✓
		ROS	Broad spectrum	Non-specific cell killer; affects wound healing; limits the application of ROS by the anaerobic environment in biofilms; instability	✓	✓	✓			✓	
	Physical methods	PTT	Long-range control; broad spectrum	Damage to normal tissue; limited depth of treatment	✓	✓	✓			✓	
		Nanomotors	Broad spectrum; improves the penetration ability of drugs	The speed and direction of MNPs are not controllable	✓		✓			✓	
	Biological method	Phages invasion	High efficiency; specificity; self-adaptability; non-toxic	Complex preparation work; induce anaphylaxis		✓			✓		✓
		The role of probiotics	Mutual restraint, security, stability	Slow effect					✓		✓

In the future, the development trend of controlling bacterial biofilms will mainly focus on the following aspects:

(1) Study of the composition and structure of biofilms: In-depth understanding of the composition and structure of bacterial biofilms is essential for controlling their development. Future research will focus on exploring molecular-level insights and the application of surface nanotechnology to further understand and intervene in the formation process of bacterial biofilms.
(2) Development of novel antibiotics and antimicrobials: Currently, there is an increasing number of bacteria that are becoming resistant to traditional antibiotics. Therefore, future research should focus on developing new natural or synthetic antibiotics and antimicrobials to effectively combat bacterial biofilms.
(3) Research and application of new sterilization techniques: In addition to traditional antimicrobial drugs, future research should further explore and apply innovative sterilization techniques and methods in nanotechnology and bioengineering.
(4) Pay close attention to bacterial biofilms in the other fields: Although bacterial biofilms are often considered as pathogens, they also pose a significant potential menace in certain fields. For example, the presence of biofilms adhered to the surfaces of food processing equipment and certain foods poses challenges in terms of cleaning, thereby elevating the potential risks associated with food safety. While the biofilms are also adhered to the instrument's surface, thereby reducing its lifespan. Additionally, the presence of biofilms in sewage poses a significant challenge to wastewater treatment processes. Furthermore, the proliferation of biofilms in livestock and feeding environments has a significant impact on the quality and cost of meat. In the future, research and exploration of controlling biofilms in other fields should be concerned.

## Declaration of competing interest

The authors declare that they have no known competing financial interests or personal relationships that could have appeared to influence the work reported in this paper.

## Data Availability

No data was used for the research described in the article.
